# Performance assessment of ontology matching systems for FAIR data

**DOI:** 10.1186/s13326-022-00273-5

**Published:** 2022-07-15

**Authors:** Philip van Damme, Jesualdo Tomás Fernández-Breis, Nirupama Benis, Jose Antonio Miñarro-Gimenez, Nicolette F. de Keizer, Ronald Cornet

**Affiliations:** 1 Amsterdam UMC location University of Amsterdam, Department of Medical Informatics, Meibergdreef 9, Amsterdam, The Netherlands; 2 Amsterdam Public Health, Digital Health & Methodology, Amsterdam, The Netherlands; 3 Departamento de Informática y Sistemas, Universidad de Murcia, IMIB-Arrixaca, Murcia, Spain; 4 Amsterdam Public Health, Methodology & Quality of Care, Amsterdam, The Netherlands

**Keywords:** Ontology matching, FAIR data, Semantic interoperability, Rare diseases

## Abstract

**Background:**

Ontology matching should contribute to the interoperability aspect of FAIR data (Findable, Accessible, Interoperable, and Reusable). Multiple data sources can use different ontologies for annotating their data and, thus, creating the need for dynamic ontology matching services. In this experimental study, we assessed the performance of ontology matching systems in the context of a real-life application from the rare disease domain. Additionally, we present a method for analyzing top-level classes to improve precision.

**Results:**

We included three ontologies (NCIt, SNOMED CT, ORDO) and three matching systems (AgreementMakerLight 2.0, FCA-Map, LogMap 2.0). We evaluated the performance of the matching systems against reference alignments from BioPortal and the Unified Medical Language System Metathesaurus (UMLS). Then, we analyzed the top-level ancestors of matched classes, to detect incorrect mappings without consulting a reference alignment. To detect such incorrect mappings, we manually matched semantically equivalent top-level classes of ontology pairs. AgreementMakerLight 2.0, FCA-Map, and LogMap 2.0 had F1-scores of 0.55, 0.46, 0.55 for BioPortal and 0.66, 0.53, 0.58 for the UMLS respectively. Using vote-based consensus alignments increased performance across the board. Evaluation with manually created top-level hierarchy mappings revealed that on average 90% of the mappings’ classes belonged to top-level classes that matched.

**Conclusions:**

Our findings show that the included ontology matching systems automatically produced mappings that were modestly accurate according to our evaluation. The hierarchical analysis of mappings seems promising when no reference alignments are available. All in all, the systems show potential to be implemented as part of an ontology matching service for querying FAIR data. Future research should focus on developing methods for the evaluation of mappings used in such mapping services, leading to their implementation in a FAIR data ecosystem.

**Supplementary Information:**

The online version contains supplementary material available at (10.1186/s13326-022-00273-5).

## Background

**Fig. 1:**
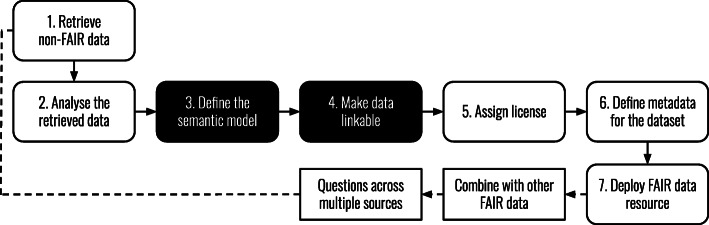
The FAIRification process. Adapted from GO FAIR [3]. This study focuses on step 3 (define the semantic model) and step 4 (make data linkable)

The generation, collection, and usage of data are crucial for scientific research. Consequently, the sharing and reuse of research data have become more and more important, leading to guidelines such as those for the European Union’s Horizon 2020 program that mandate open access to scientific publications and research data [1]. In 2016, a group of researchers and other stakeholders, with an interest in the findability and reuse of research data, published a set of principles to propagate the reuse of research data for machines and humans [2]. These principles were presented as the FAIR Guiding Principles for scientific data management and stewardship (Findable, Accessible, Interoperable, and Reusable). The process of making data FAIR is often referred to as FAIRification, which has been described in seven steps by the GO FAIR initiative (Fig. 1) [3].

When making data FAIR, a semantic model of the data needs to be defined (step 3), and the data need to be made linkable (step 4), for both of which ontologies are useful as they provide some form of consensus about some domain. Using ontologies is important for improving the semantic interoperability of data [4]. Different ontologies can contain classes describing the same domain or concepts which makes them overlap [5]. Making data interoperable becomes more challenging if researchers use different ontologies for describing the same class(es). For example, the class ‘cystic fibrosis’ is described in multiple biomedical ontologies. A systematic analysis of term overlap and term reuse across biomedical ontologies in BioPortal found an approximate overlap of over 25% and less than 9% of reuse of classes between ontologies [5]. Another paper studied the reuse of logical axioms in biomedical ontologies and discovered that 49 out of 123 ontologies did not apply any type of reuse [6]. Hence, annotated data does not mean interoperable data per se, as different ontologies would need to be matched. Ontology matching aims to make ontologies interoperable by matching semantically related classes from different ontologies, resulting in alignments between ontologies. Ontology matching can make data interoperable when data(sets) are annotated using different ontologies [7]. A specific community may or may not define standards to which the entire community should adhere, including specific ontologies. Ontology matching would then be of use to achieve interoperability within and between communities, as different communities might use different standards. The context and motivation for this study originates from the European Joint Programme on Rare Diseases (EJP RD), a large pan-European project that focuses on creating an ecosystem for rare disease research and care [8]. One of the objectives of the EJP RD is to build a FAIR-compliant data discovery platform that describes rare disease resources and enables researchers to query data from multiple resources at different locations. These sources may use different ontologies which requires ontology matching to enable querying between sources.

### Problem statement

Many matching techniques and systems have been developed but research on real-life applications has been scarce [9]. Ontology matching should contribute to FAIR data by providing interoperability between data sources that are annotated with classes from different ontologies. Matching ontologies is not a one-time task as both datasets and ontologies change over time. Hence, there is a need for dynamic ontology matching services [7]. Despite the active research community behind ontology matching, it remains unclear how matching systems and techniques would perform in the context of FAIR data. Therefore, the usefulness and performance of existing ontology matching systems should be evaluated in the light of real-life applications that follow the FAIR Guiding Principles.

### Objective and research questions

**Fig. 2:**
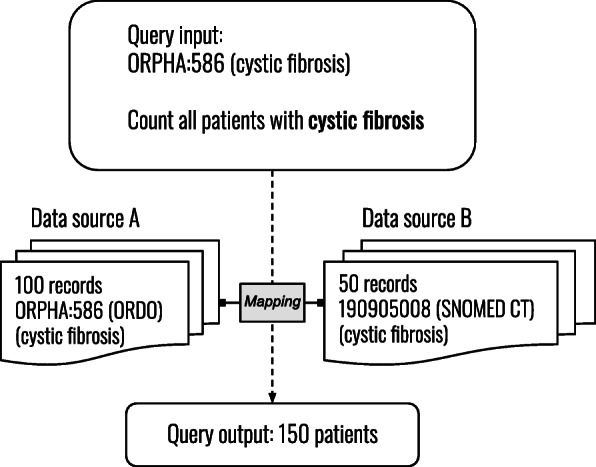
Use case for this study. How ontology matching can enable data querying of distributed data sources. The mentioned ontologies are ORDO (Orphanet Rare Disease Ontology) [44] and SNOMED Clinical Terms (SNOMED CT) [16]

This experimental study aims to measure the performance of ontology matching systems in the context of a FAIR-compliant data discovery platform, for ontologies that are relevant for the rare disease domain. By doing so, this study intends to contribute to the use of ontology matching systems for FAIR data. Figure 2 depicts how such a data discovery platform could benefit from ontology matching. For this use case, a system should also be able to determine that two matched classes belong to the same category, for example, by saying that the classes ‘cystic fibrosis’ and ‘multiple sclerosis’ are both a ‘disease’. Therefore, we analyze the top-level hierarchies of matched classes, to detect classes whose top-level ancestors are semantically equivalent. The following research questions will be discussed:

1. What is the performance of automated ontology matching systems to expose mappings between ontologies used in the rare disease research domain?
2. To what extent are currently available ontology matching systems useful for implementation in FAIR-related projects that focus on querying distributed data?

## Preliminaries

### Basic definitions

We adopt basic definitions from [10], which were modified when desired for the scope of this work. *Ontology matching* is the process of finding relationships between classes of different ontologies. Examples of relations are equivalence (≡), subsumption ($\sqsubseteq $), more generic (≥), or more specific (≤) [10]. The matching process outputs an *alignmentA* which contains mappings between classes of ontologies *O* and *O*^′^. A *mappingm* is the relationship, according to an alignment, between different classes of two ontologies. Some papers refer to mapping as correspondence. Formally a mapping can be defined as a triple by a pair of ontologies *O* and *O*^′^ and a set of mapping relations $\Theta = \{ \equiv, \sqsubseteq, \leq, \geq \}$:

$$m = \langle e,e',r \rangle $$

where *e*∈*O*,*e*^′^∈*O*^′^ and *r*∈*Θ*. Additionally, a mapping can include metadata such as a confidence value and identifiers. Equivalence mappings are of interest for this study, for instance, a matching system should be able to detect that two classes represent the same type of disease.

### Classification of ontology matching techniques

**Fig. 3:**
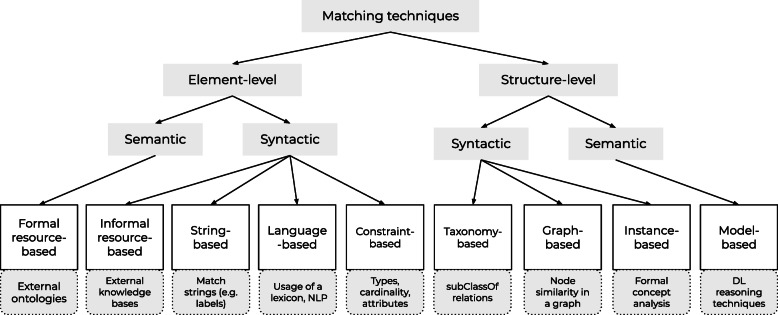
Classification of matching techniques. Adapted from [10]. For each category, an example of a possible implementation is given

Ontology matching techniques can be classified using a model that organizes techniques based on their input interpretation and granularity [10]. Figure 3 shows an adapted version of this classification model. The granularity of a matching system can be defined by two levels: elemental and structural. Element-level matching techniques focus on a class without considering its relationship to other classes, structure-level matching techniques focus on a class within the structure of the ontology. At each level, the model makes a distinction between semantic and syntactic matching techniques. Syntactic matching techniques use only the information of a class without interpretation, such as the textual labels or synonyms. Semantic matching techniques add meaning to the structural information by using a reasoner or external resources. The input of a matching system can be interpreted using nine techniques, which are mentioned in Fig. 3 including examples of implementations. The ontology matching systems used in this study apply one or more of these techniques.

## Methods

**Fig. 4:**
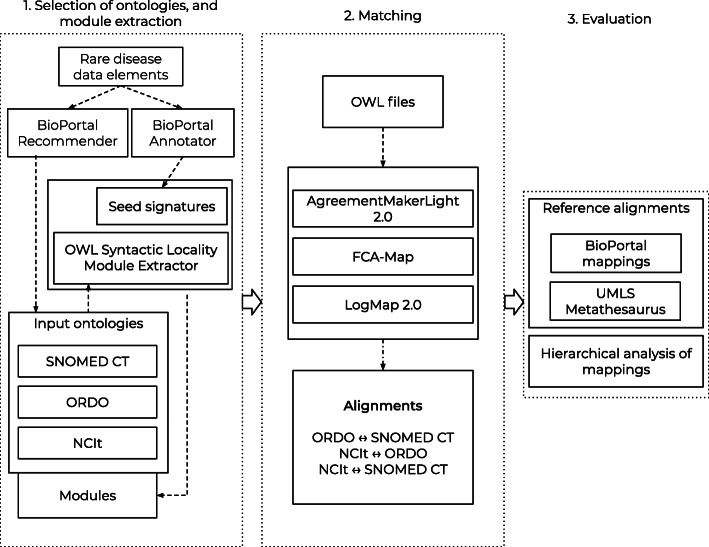
Overview of the performed experiments. Selection of ontologies, ontology module extraction, matching the ontologies using the selected matching systems, evaluating the alignments using reference alignments and an hierarchical analysis of mappings. The matching systems were selected beforehand

We performed an experimental study for measuring the performance of ontology matching systems in the context of a FAIR-compliant data discovery platform, for ontologies that are relevant for the rare disease domain. First, we evaluated the alignments generated by the matching systems using two reference alignments. Additionally, we analyzed the top-level hierarchies of classes in the mappings. The following steps were carried out: (1) selecting relevant biomedical ontologies and extracting a module; (2) generating alignments between ontology pairs; and (3) a two-part evaluation of the alignments that were obtained. Figure 4 shows an overview of the performed experiments. Development was done using Java version 8 and data analysis using R version 4.0.1 [11].

### Selection of ontologies

To select ontologies relevant for the rare disease domain, we used a set of rare disease-related keywords as input for the BioPortal Recommender [12]. For annotating its input the Recommender uses the BioPortal Annotator [13]. The input keywords were extracted from the set of common data elements for rare disease registries (items in the element and coding names columns) [14], and the classifications of rare diseases from Orphanet (all categories and one random disease per category) [15]. Items, i.e., one or multiple words, could have multiple annotations. The full list of data items can be found in the Appendix. The Recommender was run using the default configuration and the first two ontologies were selected from the list, namely SNOMED Clinical Terms (SNOMED CT, international Edition release 31-01-2020) [16], and the National Cancer Institute Thesaurus (NCIt, version 20.02d) [17]. SNOMED CT is a major biomedical ontology that contains more than 350,000 classes and includes rare disease content. NCIt also covers the biomedical domain and has more than 150,000 classes. Finally, we added the Orphanet Rare Disease Ontology (ORDO, version 2.9.1) as a third ontology as it specifically targets the rare disease domain, containing almost 15,000 classes. All ontologies were available in the Web Ontology Language (OWL) format, which is a standard maintained by the World Wide Web Consortium (W3C) [18].

### Module extraction

We extracted a module from each ontology, which is a subset of the original ontology. Modules allow to only work with relevant information from the ontology based on classes that are of interest [19]. These smaller subsets allowed us to run experiments using less computational resources and made it easier to understand and browse the structure of the ontologies. The OWL API includes a syntactic locality module extractor [20]. This module extractor uses a so-called seed signature as input to extract a subset from an ontology. This seed signature is a list of classes from the parent ontology on which the module is based. The module extractor can extract three types of modules: star, bottom, and top. A top module includes all subclasses and (sub)properties of the classes in the seed signature, a bottom module does the opposite by including the superclasses and (super)properties. A star module combines both strategies by including the intersection of the top and bottom modules. We extracted a star module from NCIt, ORDO, and SNOMED CT. The seed signatures contained the annotations of the rare disease data items as returned by the BioPortal Annotator. To ensure that modules included the entire top-level hierarchy of the original ontology, the seed signature included all ancestors of those annotated classes. In the rest of this paper the star-type module will be referred to as ‘module’.

### Matching systems and alignments

The ontology matching systems were selected from those that participated in the Ontology Alignment Evaluation Initiative (OAEI) 2019 edition [21]. The OAEI has been an annual recurring event for the performance evaluation of ontology matching systems since 2004. The event is composed of multiple tracks, each addressing various ontologies and matching tasks. Systems participating in the ‘Large Biomedical Ontologies’ and ‘Disease and Phenotype’ tracks were of particular interest, although systems from all tracks were eligible for inclusion. The Disease and Phenotype track includes a matching task with ORDO [22]. To ensure diversity among the systems in our experiment, we selected systems that implemented different matching techniques, according to the classification of Euzenat et al. (Fig. 4) [10]. Furthermore, we only selected systems whose source code was available in a public repository (to ensure we could use them). This led to the inclusion of three systems: AgreementMakerLight 2.0 (AML) [23, 24], FCA-Map [25], and LogMap 2.0 (LogMap) [26, 27]. Table 1 shows that those systems implement most matching techniques shown in Fig. 4.

**Table 1:** Classification of the matching systems based on the classification model of [10]. The systems that were used in this study are AgreementMakerLight 2.0 [24], FCA-Map [25], and LogMap 2.0 [27]

	Agreement MakerLight 2.0	FCA-Map	LogMap 2.0
**Element level**			
Semantic: Formal resource-based	X	-	-
Syntactic: Informal resource-based	-	-	-
Syntactic: String-based	X	X	X
Syntactic: Language-based	X	X	X
Syntactic: Constraint-based	-	X	-
**Structure level**			
Semantic: Model-based	-	X	X
Syntactic: Instance-based	-	X	-
Syntactic: Graph-based	-	-	X
Syntactic: Taxonomy-based	-	-	X

**Fig. 5:**
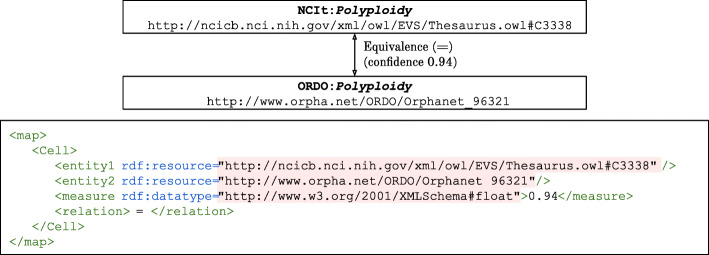
Mapping example. The class *Polyploidy* is mapped between NCIt (National Cancer Institute thesaurus [17]) and ORDO (Orphanet Rare Disease Ontology [44]). Shown are a chunk of the RDF output from the alignment and a visual representation of the mapping

The selected matching systems were run with their default configuration and no changes were made to the systems’ parameters. The output of the matching systems, the alignments, were saved in the general format provided by the Alignment API [28]. Each run was assigned 64GB of RAM. The matching systems did not need any user-input during the matching process, i.e. they provided automated ontology matching. Ontology pairs were used as input: ORDO-SNOMED CT, NCIt-ORDO, NCIt-SNOMED CT (note that matching A to B is equivalent to matching B to A). All alignments contained pairwise equivalence mappings and included the URI of each class. Figure 5 shows an example of a mapping between NCIt and ORDO.

### Hierarchical analysis of mappings

**Fig. 6:**
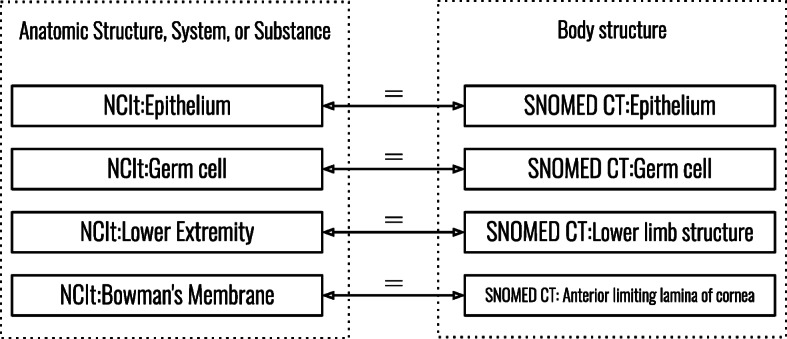
Example of a manually created top-level hierarchy mapping. The four classes from NCIt and SNOMED CT were matched by the matching system, and all four mappings were present in the reference alignments (true positive). Analyzing the top-level hierarchies reveals that NCIt classes are descendants of *Anatomic Structure, System, or Substance* and SNOMED CT classes of *Body structure*. A manual mapping between those top-level classes can then be created for NCIt-SNOMED CT

**Fig. 7:**
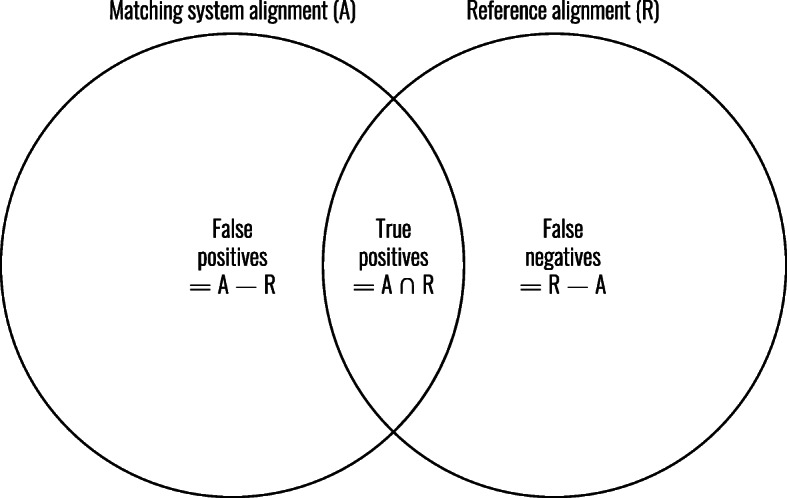
Categories for evaluation with BioPortal and UMLS Metathesaurus reference alignments. Adapted from [10]

Biomedical ontologies have a high amount of information in their lexical labels, hence, ontology matching systems often primarily use lexical matching techniques [29]. However, a mapping containing classes that originate from different top-level classes can be incorrect even if the classes have lexically similar labels. For example, two classes each labeled as *bone fracture* where the first is a descendant of the class *clinical finding* and the second of *body structure*. Each top-level hierarchy of an ontology contains classes that are of similar types, and every descendant of a top-level class shares the IS-A relation with its ancestor(s). The use case for this study considers that a query system should be able to tell that two classes from different ontologies belong to the same category. For instance, a system should be able to tell that two classes are both diseases. Therefore, we analyzed the mappings of the matching systems by comparing the top-level hierarchies of matched classes. Mappings between the top-level classes of NCIt-ORDO, NCIt-SNOMED CT, and ORDO-SNOMED CT were created manually. We created these manual mappings by inspecting the top-level hierarchies of true positive mappings (Fig. 7), based on the reference alignments. More details about the reference alignments will be given in the next section. The class descriptions were also used to determine whether or not two top-level classes should be matched. Manually matched top-level classes were considered to be semantically equivalent. Figure 6 shows an example of how such a manual mapping was created.

### Evaluation of performance

We used two reference alignments to evaluate the alignments generated by the matching systems. Alignments were evaluated against each reference alignment separately. The first reference alignment contained mappings from BioPortal and the second contained mappings based on the Unified Medical Language System Metathesaurus (UMLS) [30]. Both were chosen because they are used as reference alignments in the OAEI disease and phenotype track and large BioMed track respectively. Reference alignments for the ontology modules were derived from their full-size counterparts by removing mappings between classes which were not present in the module.

#### BioPortal

The BioPortal reference alignment was considered a baseline; an alignment that is highly incomplete in most cases [22]. The BioPortal mappings for ORDO, NCIt, and SNOMED CT are skos:closeMatch mappings based on the Lexical OWL Ontology Matcher (LOOM) [31]. LOOM is a simple string matching algorithm that compares the preferred names and synonyms of classes in both ontologies. BioPortal mappings were retrieved using the BioPortal API.

#### UMLS metathesaurus

The UMLS-based reference alignment was considered a silver standard; an alignment that is not necessarily complete or correct [22, 32]. The UMLS Metathesaurus groups all semantically equivalent classes using a code: the concept unique identifier (CUI). A single class can have multiple CUI codes. The reference alignment was extracted locally from a subset of the UMLS Metathesaurus (version 2020AA), this method was also used by Jimenéz-Ruiz et al. [33]. This subset was obtained using the MetamorphoSys tool of the UMLS by retrieving the MRCONSO.RRF file, and installed locally using MySQL Community Server version 5.6.48. Pairwise mappings were retrieved by first getting all available CUIs for every class in each ontology. Then, all classes of ontology A and B that had at least one correspondent CUI were included as a mapping in the reference alignment. ORDO is not present in the UMLS but does include CUI code mappings as annotations in the ontology. Hence, ORDO CUIs were not retrieved from the UMLS but instead directly from the ontology itself.

#### Performance metrics

We evaluated the alignments by classifying each mapping as true positive (TP, present in both the alignment as the reference alignment), false positive (FP, only present in the alignment), or false negative (FN, only present in the reference alignment) (see Fig. 7). True negatives were not included as there was no gold standard available that contained all possible correct mappings.

Subsequently, we measured performance of the matching systems by calculating precision (Eq. 1), recall (Eq. 2), and F-measure (F1-score) (Eq. 3). Precision shows the proportion of mappings in the alignment that are classified as true positive. Recall shows the proportion of mappings in the reference alignment that are also present in the alignment. F-measure combines precision and recall by calculating their harmonic mean. For precision, recall, and F-measure a score of 1 means a perfect result and 0 is the worst result. The mappings were first evaluated without and then with taking the hierarchical analysis into account. For the latter, false-positive mappings (not in the UMLS nor BioPortal) were marked as incorrect if their top-level hierarchy classes were not present in the set of manually created mappings. We recalculated precision and F-measure after discarding those incorrect mappings from the alignments. Finally, we generated consensus alignments based on majority votes and evaluated these alignments. Consensus alignments between all systems were generated by selecting mappings that were selected by two or more systems (vote ≥ 2), and three systems (vote=3). Additionally, we added consensus alignments of three combinations of matching system pairs (vote=2).

1 $$  Precision = \frac{TP}{TP + FP}  $$

2 $$  Recall = \frac{TP}{TP + FN}  $$

3 $$  \text{F-measure} = 2 \times \frac{precision \times recall}{precision + recall}  $$

## Results

### Rare disease data elements and modules

A total of 117 data items were extracted from the set of common data elements for rare disease registries and the Orphanet rare disease classifications. The BioPortal Recommender annotated 42% of the input with ORDO classes, 52% with SNOMED CT classes, and 65% with classes from NCIt. The seed signatures contained 471 classes for SNOMED CT, 74 for ORDO, and 547 for NCIt. Table 2 shows the details of the extracted modules. The modules contained between 0.4-2% of the total amount of classes and axioms of the whole ontologies.

**Table 2:** Details of the ontologies and extracted modules

	ORDO			SNOMED CT			NCIt		
Classes in module (% of total)	299 (2%)			1,408 (0.4%)			1.014 (0.7%)		
Axioms in module (% of total)	2.227 (0.9%)			7,105 (0.4%)			19,017 (0.7%)		
Object properties in module (% of total)	7 (39%)			16 (13%)			40 (41%)		
Total classes	14,502			352,449			156,172		
Total axioms	234,982			1,629,354			2,543,710		
Total object properties	18			120			97		

### Alignments

Alignments were created between the ontology pairs ORDO-SNOMED CT, NCIt-ORDO, and NCIt-SNOMED CT. A total of six OWL files were used as input for each matching system, two files per ontology (the module and whole ontology). This resulted in a total of 18 alignments, six per matching system. All alignments contained mappings with an equivalence relation. In terms of run time, AML and LogMap were the fastest, the whole ontology alignment of NCIt-SNOMED CT was created within a few hours. FCA-Map was slower and took 6-8 hours for NCIt-SNOMED CT. Table 3 shows the number of mappings per alignment for both the whole ontologies and the modules.

**Table 3:** Details of the alignments. Shown are the number of mappings in the alignments for the whole ontologies and modules

	AgreementMakerLight 2.0		FCA-Map		LogMap 2.0	
	# mappings (whole ontology)	# mappings (module)	# mappings (whole ontology)	# mappings (module)	# mappings (whole ontology)	# mappings (module)
ORDO - SNOMED CT	6,463	42	4,973	46	5,742	53
NCIt - ORDO	2,543	36	4,663	47	2,679	31
NCIt - SNOMED CT	18,887	193	26,630	220	23,885	214

### Evaluation: UMLS metathesaurus and BioPortal

Table 4 shows the number of mappings in the reference alignments that were extracted from BioPortal and the UMLS Metathesaurus. All reference alignments from the UMLS Metathesaurus contained more mappings than the ones from BioPortal. The overlap between the NCIt-ORDO and NCIt-SNOMED CT reference alignments was the largest with a weighted overlap, accounting for the difference in number of mappings per reference alignment, of 45% and 57% respectively (whole ontologies). The ORDO-SNOMED CT reference alignments had the smallest overlap, namely 14% (modules) and 25% (whole ontologies).

**Table 4:** Details of the reference alignments. Shown are the number of mappings in the BioPortal and UMLS Metathesaurus reference alignments. Also shown are the overlap and harmonic mean of the overlap between the alignments. The harmonic mean was calculated by weighting the reference alignment means by the number of mappings in each reference alignment

Ontology pair	Ontology type	Mappings UMLS	Mappings BioPortal	Overlap	Harmonic mean overlap
ORDO-SNOMED CT	Module	35	7	3	14%
NCIt-ORDO	Module	27	18	12	53%
NCIt-SNOMED CT	Module	127	90	56	52%
ORDO-SNOMED CT	Whole ontology	3,861	1,750	776	28%
NCIt-ORDO	Whole ontology	1,484	1,450	656	45%
NCIt-SNOMED CT	Whole ontology	19,309	16,290	10,195	57%

Table 5 shows the mean evaluation results for the whole ontologies. For all ontology pairs, the recall of alignments evaluated against BioPortal was higher than the recall of alignments evaluated against the UMLS. The precision of the ORDO-SNOMED CT alignment was higher when using the UMLS-based reference alignment (0.45 precision) than the BioPortal reference alignment (0.28 precision). The opposite was the case for NCIt-ORDO and NCIt-SNOMED CT, where the precision scores were higher for BioPortal than the UMLS. AML had the highest F1-score for BioPortal (0.66), all matching systems had an overall higher F1-score for BioPortal than the UMLS. LogMap had a higher precision for the UMLS (0.47) than for BioPortal (0.45). AML had a higher precision for BioPortal (0.54) than for the UMLS (0.47). NCIt-SNOMED CT had the highest recall and precision among all ontology pairs and systems.

**Table 5:** Evaluation results of the whole ontologies. Shown is the mean precision/recall/F1-score for both the UMLS and BioPortal. The scores for the ontology pairs indicate the mean of all matching systems, the scores for the matching systems indicate the mean of all ontology pairs

Pair or matching system	Precision UMLS	Precision BioPortal	Recall UMLS	Recall BioPortal	F1-score UMLS	F1-score BioPortal
ORDO - SNOMED CT	0.45	0.28	0.66	0.89	0.53	0.42
NCIt - ORDO	0.33	0.44	0.67	0.91	0.43	0.58
NCIt - SNOMED CT	0.55	0.67	0.66	0.94	0.60	0.78
AgreementMakerLight 2.0	0.47	0.54	0.66	0.96	0.55	0.66
FCA-Map	0.39	0.39	0.64	0.90	0.46	0.53
LogMap 2.0	0.47	0.45	0.69	0.88	0.55	0.58

Table 6 shows the mean results for the modules. The recall for all ontology pairs was higher for BioPortal than for the UMLS, which corresponds to the results of the whole ontologies. The UMLS precision was higher than the BioPortal precision for all ontology pairs. Overall, all matching systems had a higher F1-score for the UMLS than for BioPortal.

**Table 6:** Evaluation results of the modules. Shown is the mean precision/recall/F1-score for both the UMLS and BioPortal. The scores for the ontology pairs indicate the mean of all matching systems, the scores for the matching systems indicate the mean of all ontology pairs

Pair or matching system	Precision UMLS	Precision BioPortal	Recall UMLS	Recall BioPortal	F1-score UMLS	F1-score BioPortal
ORDO - SNOMED CT	0.45	0.14	0.60	0.95	0.51	0.25
NCIt - ORDO	0.49	0.47	0.67	0.96	0.56	0.62
NCIt - SNOMED CT	0.51	0.42	0.84	0.98	0.64	0.59
AgreementMakerLight 2.0	0.49	0.37	0.67	0.97	0.57	0.51
FCA-Map	0.42	0.31	0.68	1.00	0.52	0.46
LogMap 2.0	0.53	0.35	0.77	0.92	0.62	0.49

Results per matching system (i.e., all ontology pairs) and ontology pair (i.e., all matching systems) are averaged for easier interpretation and because individual differences were, in general, small. See Additional File 1 for all individual results.

### Evaluation using the hierarchical analysis of mappings

Table 7 shows the manually created top-level hierarchy mappings. We created three mappings between the top-level classes of ORDO-SNOMED CT, six mappings for NCIt-ORDO, and 13 mappings for NCIt-SNOMED CT. Table 8 shows the results of the analysis. On average 10% (whole ontologies) of the mappings in an alignment contained classes of which the top-level hierarchies were not present in the manual top-level mappings set. Mappings that were true positive for either BioPortal and/or the UMLS were kept in the alignments. On average, 4.6% of the mappings in the alignments contained classes of which the top-level hierarchy was not present in the manual mappings set and were false positive. The module alignments had an average of 19% of incorrect hierarchy mappings, and 8.7% of the mappings in the alignments were false positives with an incorrect top-level hierarchy.

**Table 7:** Manually created mappings of top-level classes

ORDO	SNOMED CT
clinical entity (Orphanet_C001)	Clinical finding (404684003)
genetic material (Orphanet_C010)	Substance (105590001)
geography (Orphanet_C009)	Environment or geographical location (308916002)
NCIt	ORDO
Disease, Disorder or Finding (C7057)	clinical entity (Orphanet_C001)
Gene Product (C26548)	genetic material (Orphanet_C010)
Conceptual Entity (C20181)	geography (Orphanet_C009)
Conceptual Entity (C20181)	inheritance (Orphanet_C005)
Property or Attribute (C20189)	age of onset (Orphanet_C023)
NCIt	SNOMED CT
Anatomic Structure, System, or Substance (C12219)	Body structure (123037004)
Disease, Disorder or Finding (C7057)	Clinical finding (404684003)
Property or Attribute (C20189)	Qualifier value (362981000)
Anatomic Structure, System, or Substance (C12219)	Substance (105590001)
Activity (C43431)	Procedure (71388002)
Organism (C14250)	Organism (410607006)
Drug, Food, Chemical or Biomedical Material (C1908)	Substance (105590001)
Drug, Food, Chemical or Biomedical Material (C1908)	Pharmaceutical / biologic product (373873005)
Manufactured Object (C97325)	Physical object (260787004)
Property or Attribute (C20189)	Observable entity (363787002)
Conceptual Entity (C20181)	Environment or geographical location (308916002)
Conceptual Entity (C20181)	Social context (48176007)
Conceptual Entity (C20181)	Observable entity (363787002)

**Table 8:** Hierarchy analysis results. The number of mappings whose classes’ top-level ancestors were not matched manually (Table 7) are shown for each system and ontology pair. The amount and percentage of false positives (FP) refer to the mappings that were discarded from the alignment for recalculation of both the precision and F1-score

		Whole ontology		Module	
Matching system	Ontology pair	Incorrect hierarchy mappings (of which FP)	Proportion of total alignment (FP)	Incorrect hierarchy mappings (of which FP)	Proportion of total alignment (FP)
AgreementMaker Light 2.0	ORDO-SNOMED CT	494 (318)	8% (5%)	9 (6)	21% (14%)
FCA-Map	ORDO-SNOMED CT	489 (310)	10% (6%)	11 (8)	24% (17%)
LogMap 2.0	ORDO-SNOMED CT	193 (106)	3% (2%)	5 (3)	9% (6%)
AgreementMaker Light 2.0	NCIt-SNOMED CT	3,055 (252)	16% (1%)	46 (13)	24% (7%)
FCA-Map	NCIt-SNOMED CT	6,868 (3,299)	26% (12%)	60 (23)	27% (10%)
LogMap 2.0	NCIt-SNOMED CT	3,790 (1,180)	16% (5%)	42 (9)	20% (4%)
AgreementMaker Light 2.0	NCIt-ORDO	127 (102)	5% (4%)	4 (1)	11% (3%)
FCA-Map	NCIt-ORDO	1,229 (1,170)	3% (3%)	12 (8)	26% (17%)
LogMap 2.0	NCIt-ORDO	130 (92)	5% (3%)	3 (0)	10% (0%)

The results of the whole ontologies for recalculating the precision and F1-score, after discarding false positive mappings with an incorrect top-level hierarchy, are shown in Table 9. Precision and F1-score values increased between 0.01 and 0.05, for all ontology pairs and matching systems. The exception was FCA-Map, for which the BioPortal precision increased from 0.39 to 0.45 (+0.06). Table 10 shows the new precision and F1-scores for the modules. The scores increased between 0 and 0.06 points overall, except for the NCIt-SNOMED CT precision and F1-score for BioPortal (+0.29 and +0.22 respectively).

**Table 9:** Evaluation results of the whole ontologies after removing false positive mappings with an incorrect top-level hierarchy. Shown is the mean precision/F1-score for both the UMLS and BioPortal. Ontology pairs indicate the mean of all matching systems, matching systems indicate the mean of all ontology pairs. Recall has not changed and is therefore not included

Pair or matching system	Precision UMLS	Precision BioPortal	F1-score UMLS	F1-score BioPortal
ORDO - SNOMED CT	0.47 (+0.02)	0.29 (+0.01)	0.55 (+0.02)	0.44 (+0.02)
NCIt - ORDO	0.36 (+0.03)	0.48 (+0.04)	0.46 (+0.03)	0.62 (+0.04)
NCIt - SNOMED CT	0.59 (+0.04)	0.71 (+0.04)	0.62 (+0.02)	0.81 (+0.03)
AgreementMakerLight 2.0	0.49 (+0.02)	0.56 (+0.02)	0.56 (+0.01)	0.68 (+0.02)
FCA-Map	0.44 (+0.05)	0.45 (+0.06)	0.51 (+0.05)	0.59 (+0.06)
LogMap 2.0	0.48 (+0.01)	0.47 (+0.02)	0.56 (+0.01)	0.60 (+0.02)

**Table 10:** Evaluation results of the modules after removing false-positive mappings with an incorrect top-level hierarchy. Shown is the mean precision/F1-score for both the UMLS and BioPortal. Ontology pairs indicate the mean of all matching systems, matching systems indicate the mean of all ontology pairs. Recall has not changed and is therefore not included

Pair or matching system	Precision UMLS	Precision BioPortal	F1-score UMLS	F1-score BioPortal
ORDO - SNOMED CT	0.51 (+0.06)	0.16 (+0.02)	0.55 (+0.04)	0.28 (+0.03)
NCIt - ORDO	0.52 (+0.03)	0.50 (+0.03)	0.58 (+0.02)	0.65 (+0.02)
NCIt - SNOMED CT	0.59 (+0.08)	0.71 (+0.29)	0.62 (+0.02)	0.81 (+0.22)
AgreementMakerLight 2.0	0.54 (+0.05)	0.39 (+0.02)	0.59 (+0.02)	0.52 (+0.01)
FCA-Map	0.49 (+0.07)	0.37 (+0.06)	0.57 (+0.05)	0.52 (+0.06)
LogMap 2.0	0.55 (+0.02)	0.36 (+0.01)	0.64 (+0.02)	0.49 (+0.00)

### Consensus alignments

Table 11 shows F1-scores of the consensus alignments based on majority votes. Precision and recall scores are included in Additional File 1. Consensus alignments among all systems containing mappings having two or more votes led to an increase in overall performance. Alignments containing mappings with vote=3 increased all F1-scores further, except UMLS scores of NCIt-SNOMED CT and ORDO-SNOMED CT, compared to the mean scores in Table 5. When voting among matching system pairs (only those mappings selected by both systems, vote=2), pairing AML 2.0 and FCA-Map increased performance up to 0.03 points compared to the consensus alignments with three votes among all systems. F1-scores based on the UMLS for NCIt-SNOMED CT and ORDO-SNOMED CT increased for the consensus alignment between FCA-Map and LogMap 2.0, compared to Table 5. Removing false-positive mappings with an incorrect top-level hierarchy from the consensus alignments did not increase F1-scores by more than 0.01 point.

**Table 11:** Consensus alignment results. Shown are the F1-scores for vote-based consensus alignments. The number of votes represents how many systems selected the same mapping. F1-scores when corrected for positive mappings with an incorrect top-level hierarchy are shown in parenthesis. AgreementMakerLight 2.0 is abbreviated as AML 2.0

	All systems (vote ≥ 2)	All systems (vote = 3)	AML 2.0 + FCA-Map	AML 2.0 + LogMap 2.0	FCA-Map + LogMap 2.0
**NCIt - SNOMED CT**					
F1-score BioPortal (top-level hierarchy)	0.80 (0.81)	0.87 (0.87)	0.90 (0.90)	0.87 (0.87)	0.77 (0.78)
F1-score UMLS (top-level hierarchy)	0.63 (0.65)	0.59 (0.59)	0.59 (0.59)	0.59 (0.59)	0.63 (0.64)
**NCIt - ORDO**					
F1-score BioPortal (top-level hierarchy)	0.71 (0.71)	0.79 (0.79)	0.80 (0.80)	0.73 (0.73)	0.76 (0.76)
F1-score UMLS (top-level hierarchy)	0.53 (0.54)	0.53 (0.53)	0.53 (0.53)	0.55 (0.55)	0.51 (0.52)
**ORDO - SNOMED CT**					
F1-score BioPortal (top-level hierarchy)	0.44 (0.44)	0.49 (0.49)	0.50 (0.50)	0.44 (0.44)	0.47 (0.47)
F1-score UMLS (top-level hierarchy)	0.56 (0.57)	0.51 (0.51)	0.52 (0.52)	0.55 (0.56)	0.52 (0.52)

## Discussion

We evaluated the performance of three existing ontology matching systems using reference alignments based on the UMLS and BioPortal. Additionally, we analyzed the top-level hierarchies of mappings using manually created mappings between the top-level classes of ontology pairs. These experiments should contribute to the use case of querying distributed data sources, in the context of FAIR data.

### Principal findings

***What is the performance of automated ontology matching systems to expose mappings between ontologies used in the rare disease research domain?***

The systems exposed, on average, 5.726 mappings between ORDO-SNOMED CT, 3.295 mappings between NCIt-ORDO, and 23.134 mappings between NCIt-SNOMED CT. Obtained F1-scores were 0.55/0.66 (AML, UMLS/BioPortal), 0.46/0.53 (FCA-Map), and 0.55/0.58 (LogMap). The results obtained for the modules were comparable to those of the whole ontologies. As there was no gold standard available, the systems’ overall low precision (between 0.39-0.54) and high recall (between 0.64-0.96) suggests that (automatically) evaluating the correctness of mappings is indeed challenging. The systems retrieved most mappings in the reference alignments, but also exposed many additional mappings, hence the lower precision. Both reference alignments were known to be incomplete (silver standard and baseline), further research will be needed to assess whether additional mappings returned by the systems are correct. The results of the OAEI 2019 Large BioMed track (SNOMED CT-NCIt large fragment task [34]) are the closest to use as reference for interpreting performance, as the other tracks and tasks of the OAEI use other ontologies or reference alignments. Using a UMLS-based reference alignment (inconsistent mappings were flagged to be ignored), AML obtained an F1-score of 0.76, FCA-Map 0.65, and LogMap 0.71. Those OAEI results are better, although it is not a one-to-one comparison due to different reference alignment and ontology versions. Moreover, the OAEI reports using a large fragment of SNOMED CT, resulting in fewer mappings than when using the whole ontology (18,887 vs. 14,200 by AML).

Using consensus alignments (i.e., mappings selected by multiple systems) improved performance across the board (Table 11 and Additional File 1). As one would expect, selecting a higher number of votes (vote=3, mappings selected by all systems) resulted in higher precision and lower recall. In practice, one could prioritize precision over recall or vice versa for a specific application, and the ability to select a consensus alignment that fits those needs can be useful.

***To what extent are currently available ontology matching systems useful for implementation in FAIR-related projects that focus on querying distributed data?***

All systems were able to generate alignments without user intervention, which is important for data querying. Run times varied from minutes up to a few hours, depending on the size of the input ontologies. Matching systems exposed equivalence relations between classes of ontologies pairs. The use case depicted in Fig. 2 requires equivalence mappings and automated matching. This means that the application of AML, FCA-Map, and/or LogMap, in the context of the use case would be a sensible decision. However, in the case of a matching service for querying data, high precision is more important than high recall, hence the need for additional work on validating the correctness of mappings. All systems support OWL ontologies as input and export alignments as a machine-readable RDF-file. This allows joining alignments from several matching systems. Besides, analyzing top-level hierarchies of matched classes was shown to be effective in revealing mappings with classes from the same hierarchy. Table 8 shows that on average 10% of mappings had an incorrect top-level hierarchy; 90% were mappings whose top-level hierarchies matched using the manually created mappings. For example, consider a query: ‘count all patients with a rare disease’, then the hierarchical analysis can reveal that ‘cystic fibrosis’ is a rare disease and its records should be counted. The hierarchical analysis can be used in situations where no reference alignments are available. Finally, the use of modules helps for faster development and testing of matching techniques, in comparison to working with the whole ontologies. Using modules instead of the whole ontologies could be considered if speed or resources are important factors. Additionally, modularization can be used as a type of structure-level matching by removing content from the ontology that is not relevant for the application [10]. As modularization removes content from the ontologies it should be noted that this could improve or worsen the results of matching systems that use structural matching techniques, although we did not test this hypothesis.

### Strengths and limitations

Several strengths and limitations can be identified. A strength of this study is its practical approach to ontology matching using a FAIR data use case, using ontologies relevant for the rare disease domain. A limitation is that AML, FCA-Map, and LogMap are not the only matching systems available, although they cover most of the matching techniques as specified by the classification model defined by Euzenat et al. [10]. Particularly, systems that leverage machine-learning techniques were not included. Likewise, other ontologies exist that would be of use in the rare disease domain, especially considering the large number of ontologies present in, among other repositories, BioPortal. The BioPortal Annotator and Recommender were used to select the ontologies and create the seed signatures, but other similar tooling could also be used.

Another strength is the use of modules based on seed signatures derived from rare disease data elements. The smaller modules made it easier to assess the mappings manually while performing the experiments. Also, it shows potential for implementation in matching services where it is desirable to work with smaller chunks of large ontologies, e.g. for faster run times. However, since the list of rare disease data elements was not validated, it was not possible to draw any additional conclusions from the modules versus whole ontology results. For instance, we did not know if the classes contained in the modules were the most relevant ones for use in the rare disease domain.

#### Evaluation with BioPortal and UMLS reference alignments

Precision, recall, and F1-score were used as performance measures because they are widely known and used by the OAEI. However, both precision and recall introduce a problem when used in the context of ontology matching that should be mentioned. As stated by [35], both precision and recall are set-theoretic measures that do not discriminate between mappings that may be semantically equivalent but not identical. Thus, when a mapping is not present in the reference alignment it is per definition considered to be incorrect (false positive). Semantic precision and recall could solve this problem by considering mappings that are, semantically speaking, close to a mapping in the reference alignment. For example, when a mappings’ class is a super- or subclass of a correspondent class in the reference alignment.

To our knowledge, the UMLS and BioPortal based reference alignments were the only ones available that offered mappings between a wide variety of ontologies, including SNOMED CT, ORDO, and NCIt. We consider using two reference alignments for the evaluation a strength, as both contain different mappings despite their overlap of 28 to 57% (Table 4). The BioPortal mappings were considered to be a baseline alignment, as previously mentioned by the OAEI [22]. The precision and recall for BioPortal were both the highest for AML (0.54 and 0.96 respectively, whole ontology). This corresponds to the fact that both AML and BioPortal (LOOM) base their mappings on lexical techniques only. On the other hand, the mappings derived from the UMLS were considered to be a silver standard since the Metathesaurus is being maintained by domain experts. A limitation of the UMLS reference alignment used for this study is the CUI codes from ORDO, as ORDO is not included in the UMLS those CUIs were extracted from ORDO itself. The BioPortal F1-scores for the modules were 15% lower on average than the whole ontologies, which could be due to the low number of mappings in the reference alignments. Finally, earlier research mentioned that the UMLS reference alignment contains incoherent mappings [33], namely mappings that contain logical errors following from the union of the input ontologies and the mappings set [36]. Moreover, logical incoherences were also found in BioPortal mappings [37]. Such mappings were not removed and/or examined during the evaluation performed in this study.

#### Hierarchical analysis of mappings

Discarding false positive mappings, whose top-level hierarchy classes were not manually matched, did not result in much higher precision scores (up to 0.06 points higher). Nonetheless, analyzing top-level hierarchies of matched classes can be of value when applying ontology matching for FAIR data. First of all, hierarchies can exploit information about the origin of a class. For instance, if ‘pneumonia’ and ‘asthma’ are both part of the ‘disease’ hierarchy, they can be classified as such, even if it remains unknown if the classes themselves can be used interchangeably due to the lack of a reference alignment. This can be useful when querying data over multiple sources (example Fig. 2). Additionally, some classes were present in multiple mappings (a class mapped to multiple other classes), and in such cases the hierarchy analysis was able to detect incorrect mappings.

Our list of manual mappings between top-level classes may not be complete, which is a limitation of the hierarchical analysis. In addition, our method needs top-level classes to be manually matched and thus cannot be done automatically. However, even large ontologies tend to have few top-level classes which (e.g., SNOMED CT and NCIt both have 19 top-level classes). Table 12 shows four potentially incorrect mappings (as returned by the matching systems) and their top-level hierarchies. The first mapping in Table 12 is *Soft tissue* and *Disorder of soft tissue*, the first refers to the anatomic structure of soft tissue, the second refers to a disorder of this soft tissue. The second example is *Aneurysmal Bone Cyst* and *Aneurysmal bone cyst*, in which the labels are lexically identical. However, the first refers to the disease and the second to the body structure. The last example is *Cell Proliferation* matched with *Hyperplasia*, the top-level hierarchies reveal that the first class is a *Biological Process* and the second a *Body structure*. Now, this example amplifies the importance of the evaluation of the mappings by domain experts. Hyperplasia is the result of cell proliferation [38], thus the mapping could be considered correct depending on the application. Moreover, after manual inspection of the alignments, we found true positive mappings whose top-level hierarchy was incorrect according to our manual mappings. Those mappings were not flagged as incorrect because they were included in either one of the reference alignments. Yet, those mappings could suggest incorrect mappings in the reference alignment, which is out of the scope of this work.

**Table 12:** Four examples of mappings (NCIt-SNOMED CT) that are potentially incorrect based on their top-level hierarchies

#	Label class A	Label class B	Top level hierarchy class A	Top level hierarchy class B
1	Soft tissue	Disorder of soft tissue (disorder)	Anatomic Structure, System, or Substance	Clinical finding (finding)
2	Aneurysmal Bone Cyst	Aneurysmal bone cyst (morphologic abnormality)	Disease, Disorder or Finding	Body structure (body structure)
3	Abnormality	Abnormal (qualifier value)	Disease, Disorder or Finding	Qualifier value (qualifier value)
4	Cell Proliferation	Hyperplasia (morphologic abnormality)	Biological Process	Body structure (body structure)

#### Consensus alignments

We have yielded better results using consensus alignments compared to individual alignments. Consensus alignments have also been used by certain tracks of the OAEI [22, 39]. Harrow et al. mention that consensus alignments only compare how matching systems perform against each other. False positives are still likely to occur as more than one system can find the same, incorrect, mapping. Furthermore, correct mappings may only be found by one system and, thus, would not be included in a consensus alignment.

### Relation to other work

The evaluation of the ontology matching systems relates to earlier research on matching disease and phenotype ontologies, and large biomedical ontologies (Large BioMed), both of which are tracks of the OAEI [22, 40]. In addition to the BioPortal baseline reference alignments, a consensus alignment based on a voting mechanism (multiple systems returning the same mapping), and manually curated mappings, were used to evaluate the matching systems. The large biomedical ontologies track of the OAEI uses the UMLS as reference alignment, which is based on an earlier work that extracted pairwise mappings from the UMLS Metathesaurus [33]. Moreover, our work relates to a paper published in 2020 which presented a generic workflow for making data FAIR [41]. Ontology matching systems should be included in the FAIRification workflow when dealing with multiple ontologies and some form of automated matching of classes is desired.

**Table 13:** Rare disease data items. 117 in total. Item are extracted from the common data elements for rare diseases [14], and the Orphanet rare disease classifications [15]

Data items 1-31	Data items 32-62	Data items 63-93	Data items 94-117
Pseudonym	Consent to the reuse of data	Thoracic malformation	Rare Infectious Diseases
Personal information	Biological sample	Rare Urogenital Diseases	Cholera
Date of birth	Link to a biobank	Urogenital tract malformation	Rare Intoxications
Date	Biobank	Rare Surgical Thoracic Diseases	Radiation myelitis
Female	Disability	Thoracic outlet syndrome	Rare Gynaecological And Obstetric Diseases
Male	Classification of functioning	Rare Skin Diseases	Vaginal carcinoma
Foetus	Classification of disability	Ichthyosis	Rare Surgical Maxillo-facial Diseases
Sex	Disability profile	Rare Renal Diseases	Cleft palate
Patient status	Disability score	Multicystic dysplastic kidney	Rare Allergic Disease
Alive	Rare diseases	Rare Eye Diseases	Acquired angioedema
Dead	Rare Cardiac Diseases	Retinoblastoma	Rare Teratologic Disorders
Lost in follow-up	Rare cardiomyopathy	Rare Endocrine Diseases	Infectious embryofetopathy
Opted-out	Rare Developmental Anomalies During Embryogenesis	Neuroendocrine neoplasm	Chromosomal Anomalies Sorted By Chromosomes
Opt-out	Hydrops fetalis	Rare Haematological Diseases	Polyploidy
Date of death	Rare Cardiac Malformations	Mastocytosis	Rare Rheumatologic Diseases Of Childhood
Care pathway	Congenital pericardium anomaly	Rare Immunological Diseases	Kawasaki disease
First contact with specialised centre	Rare Sucking Swallowing Disorders	Graft versus host disease	Rare Disorders Potentially Indicated For Transplant
Disease history	Stickler syndrome	Rare Systemic And Rhumatological Diseases	Systemic primary carnitine deficiency
Age at onset	Rare Inborn Errors Of Metabolism	Hereditary angioedema	Prevalence
Antenatal	MPI-CDG	Rare Odontological Diseases	Cases/families
At birth	Rare Gastroenterological Diseases	Bruck syndrome	Case
Age at diagnosis	Eosinophilic gastroenteritis	Rare Circulatory System Diseases	Worldwide
Diagnosis	Rare Genetic Diseases	Congenital renal artery stenosis	Validated
Diagnosis of the rare disease	Noonan syndrome	Rare Bone Diseases	Geographic
Genetic diagnosis	Rare Neurological Diseases	Aneurysmal bone cyst	
Undiagnosed case	Spinal cord injury	Rare Otorhinolaryngological Diseases	
Phenotype	Rare Abdominal Surgical Diseases	Familial nasal acilia	
Genotype	Adenoma of pancreas	Rare Infertility	
Research	Rare Hepatic Diseases	Tuberculosis	
Patient permission	Rare vascular liver disease	Rare Neoplastic Diseases	
Agreement to be contacted for research purposes	Rare Respiratory Diseases	Germ cell tumor	

### Future research

We explored the use of ontology matching systems for a use case in the context of FAIR data and showed that existing ontology matching systems have the potential to be implemented in such environments. A problem that has not yet been solved is how mappings can be evaluated as useful or correct for their specific application. Obtaining complete reference alignments is a challenging task and such alignments are not readily available. The (automatic) evaluation of mappings for usage in an on-the-fly matching service within a FAIR data environment will become important. Therefore, future research should focus on developing methods for evaluating mappings that can be used by such matching services. Moreover, situations where no reference alignments are available should be considered. Those developments should be driven by specific use cases. Adding to this, it would be beneficial to include top-level hierarchy analysis as an additional method to ontology matching systems. Future research could focus on how to integrate this method in existing (modular) systems and workflows. For example, AgreementMaker offers an extensible architecture which may enable the inclusion of our method. We should acknowledge that additional matching methods, utilizing the structure or logic of ontologies, are not limited to top-level hierarchies. Future research could focus on discovering and analyzing other methods that have not yet been implemented by existing matching systems. Lastly, our experiment did not include matching systems based on a machine learning approach. Earlier research has demonstrated that an approach based on representation learning is effective at ontology matching [42]. Hence, investigating machine learning-based systems could be of added value. The recently added machine learning extension to the Matching and EvaLuation Toolkit (MELT) framework could aid such efforts [43]. MELT also offers so-called filters, one of which is a classifier that can be trained to classify a mapping as correct or incorrect. Such a filter could be used to improve the precision of an alignment, given it can be trained with positive and negative mappings. For the latter, a gold standard is required or negative mappings need to be created manually.

## Conclusions

We explored the performance of ontology matching systems for ontologies used in the rare disease domain, and analyzed top-level hierarchies of mappings in the context of a FAIR data use case. Our results showed that all three systems (AgreementMakerLight 2.0, FCA-Map, LogMap 2.0) were able to automatically produce modestly accurate mappings, according to our evaluation. We found that evaluating the performance of the systems is challenging, as correct and complete reference alignments are not always available. Incomplete or incorrect reference alignments impedes the evaluation of mappings, which could consequently limit the reliability of a system that queries distributed data. We presented a hierarchical analysis of mappings that seems to be promising in such situations, as it does not require a reference alignment. All in all, this work should spark interest in implementing the demonstrated ontology matching systems and top-level hierarchy analysis in a dynamic service for querying FAIR data.

## Appendix

## Supplementary Information

**Additional file 1** Individual evaluation results. Full results of the evaluation. Tables show individual results per matching system, ontology pair, ontology type (module or whole ontology). In addition, the file includes tables with individual precision and recall scores of the consensus alignments.

## Data Availability

The datasets supporting the conclusions of this article are available in the GitHub repository, https://github.com/PhilipvD/2021-jbiomedsem-ontology-matching-paper.

## Abbreviations

AML: AgreementMakerLight 2.0
CUI: Concept Unique Identifier
EJP RD: European Joint Programme on Rare Diseases
FAIR: Findable, Accessible, Interoperable, and Reusable
FCA: Formal Concept Analysis
NCIt: National Cancer Institute Thesaurus
OAEI: Ontology Alignment Evaluation Initiative
ORDO: Orphanet Rare Disease Ontology
SNOMED CT: SNOMED Clinical Terms
UMLS: Unified Medical Language System Metathesaurus
