# Supplementary material for: Performance assessment of ontology matching systems for FAIR data
Source: J Biomed Semantics. 2022 Jul 15;13:19. doi: 10.1186/s13326-022-00273-5 (PMC9284868; doi:10.1186/s13326-022-00273-5)
Supplement: Supplementary file 1 — Additional file 1 Individual evaluation results. Full results of the evaluation. Tables show individual results per matching system, ontology pair, ontology type (module or whole ontology). In addition, the file includes tables with individual precision and recall scores of the consensus alignments. [file 13326_2022_273_MOESM1_ESM.pdf]

Additional file 1 - Individual evaluation results

Each table contains the matching system(s), ontology pair, and type (module or whole ontology) used for the experiment. The mentioned ontologies are the National Cancer Institute thesaurus (NCIt), the Orphanet Rare Disease Ontology (ORDO), and SNOMED Clinical Terms (SNOMED CT). Mean results in the manuscript are based on Table (whole ontologies) and Table (modules). For example, the mean precision for ORDO-SNOMED CT (whole) using the BioPortal-based reference alignment was calculated by taking its result from each system and dividing it by three $((0.46 + 0.48 + 0.47)/3 = 0.47)$.

Table 1: *Results per matching system and ontology pair, whole ontologies.* Shown are the precision, recall, and F1-scores for the evaluation with both the BioPortal-based and UMLS-based reference alignments.

Matching system	Ontology pair	Ontology type	Precision UMLS	Precision BioPortal	Recall UMLS	Recall BioPortal	F1-score UMLS	F1-score BioPortal
AgreementMakerLight 2.0	NCIt-ORDO	Whole	0.42	0.57	0.69	0.96	0.52	0.71
AgreementMakerLight 2.0	NCIt-SNOMED CT	Whole	0.60	0.82	0.58	0.94	0.59	0.88
AgreementMakerLight 2.0	ORDO-SNOMED CT	Whole	0.46	0.28	0.72	0.98	0.56	0.44
FCA-Map	NCIt-ORDO	Whole	0.27	0.37	0.63	0.88	0.37	0.52
FCA-Map	NCIt-SNOMED CT	Whole	0.58	0.67	0.70	0.97	0.63	0.79
FCA-Map	ORDO-SNOMED CT	Whole	0.48	0.32	0.58	0.85	0.52	0.47
LogMap 2.0	NCIt-ORDO	Whole	0.39	0.50	0.68	0.89	0.49	0.64
LogMap 2.0	NCIt-SNOMED CT	Whole	0.58	0.64	0.69	0.90	0.63	0.75
LogMap 2.0	ORDO-SNOMED CT	Whole	0.47	0.27	0.69	0.85	0.56	0.41

Table 2: *Results per matching system and ontology pair, ontology modules.* Shown are the precision, recall, and F1-scores for the evaluation with both the BioPortal-based and UMLS-based reference alignments.

Matching system	Ontology pair	Ontology type	Precision UMLS	Precision BioPortal	Recall UMLS	Recall BioPortal	F1-score UMLS	F1-score BioPortal
AgreementMakerLight 2.0	NCIt-ORDO	Module	0.51	0.49	0.67	0.94	0.58	0.64
AgreementMakerLight 2.0	NCIt-SNOMED CT	Module	0.54	0.49	0.76	0.98	0.63	0.65
AgreementMakerLight 2.0	ORDO-SNOMED CT	Module	0.56	0.19	0.57	1.00	0.56	0.33
FCA-Map	NCIt-ORDO	Module	0.46	0.46	0.67	1.00	0.55	0.63
FCA-Map	NCIt-SNOMED CT	Module	0.57	0.46	0.88	1.00	0.69	0.63
FCA-Map	ORDO-SNOMED CT	Module	0.45	0.18	0.49	1.00	0.47	0.31
LogMap 2.0	NCIt-ORDO	Module	0.58	0.55	0.67	0.94	0.62	0.69
LogMap 2.0	NCIt-SNOMED CT	Module	0.55	0.42	0.89	0.96	0.68	0.58
LogMap 2.0	ORDO-SNOMED CT	Module	0.52	0.12	0.74	0.86	0.61	0.21

Table 3: *Consensus alignments, whole ontologies.* Shown are the precision, recall, and F1-scores when evaluating consensus mappings based on votes. The number of votes represents how many systems selected the same mapping. AgreementMakerLight 2.0 is abbreviated as AML 2.0.

	All systems (vote $\geq$ 2)	All systems (vote = 3)	AML 2.0 + FCA-Map	AML 2.0 + LogMap 2.0	FCA-Map + LogMap 2.0
**NCIt-SNOMED CT**					
Precision BioPortal	0.69	0.87	0.87	0.86	0.68
Precision UMLS	0.59	0.64	0.62	0.64	0.60
Recall BioPortal	0.95	0.87	0.93	0.88	0.89
Recall UMLS	0.69	0.54	0.56	0.55	0.70
F1-score BioPortal	0.80	0.87	0.90	0.87	0.77
F1-score UMLS	0.63	0.59	0.59	0.59	0.63
**NCIt-ORDO**					
Precision BioPortal	0.58	0.76	0.75	0.62	0.70
Precision UMLS	0.44	0.51	0.50	0.47	0.47
Recall BioPortal	0.91	0.83	0.86	0.86	0.85
Recall UMLS	0.67	0.55	0.56	0.65	0.60
F1-score BioPortal	0.71	0.79	0.80	0.73	0.76
F1-score UMLS	0.53	0.53	0.53	0.55	0.51
**ORDO-SNOMED CT**					
Precision BioPortal	0.29	0.35	0.35	0.30	0.33
Precision UMLS	0.48	0.50	0.49	0.49	0.49
Recall BioPortal	0.90	0.80	0.85	0.85	0.81
Recall UMLS	0.68	0.52	0.54	0.64	0.54
F1-score BioPortal	0.44	0.49	0.50	0.44	0.47
F1-score UMLS	0.56	0.51	0.52	0.55	0.52

Table 4: *Consensus alignments, whole ontologies (with top-level hierarchy filter).* Shown are the precision and F1-scores when evaluating consensus mappings based on votes, corrected for positive mappings with an incorrect top-level hierarchy. The number of votes represents how many systems selected the same mapping. AgreementMakerLight 2.0 is abbreviated as AML 2.0.

	All systems (vote $\geq$ 2)	All systems (vote = 3)	AML 2.0 + FCA-Map	AML 2.0 + LogMap 2.0	FCA-Map + LogMap 2.0
**NCIt-SNOMED CT**					
Precision BioPortal	0.71 (+0.02)	0.87 (+0.00)	0.88 (+0.01)	0.87 (+0.01)	0.70 (+0.02)
Precision UMLS	0.61 (+0.02)	0.64 (+0.00)	0.63 (+0.01)	0.64 (+0.00)	0.62 (+0.02)
F1-score BioPortal	0.81 (+0.01)	0.87 (+0.00)	0.90 (+0.00)	0.87 (+0.00)	0.78 (+0.00)
F1-score UMLS	0.65 (+0.02)	0.59 (+0.00)	0.59 (+0.00)	0.59 (+0.00)	0.64 (+0.01)
**NCIt-ORDO**					
Precision BioPortal	0.59 (+0.01)	0.76 (+0.00)	0.76 (+0.01)	0.62 (+0.00)	0.70 (+0.00)
Precision UMLS	0.45 (+0.01)	0.51 (+0.00)	0.51 (+0.01)	0.48 (+0.01)	0.48 (+0.01)
F1-score BioPortal	0.71 (+0.00)	0.79 (+0.00)	0.80 (+0.00)	0.73 (+0.00)	0.76 (+0.00)
F1-score UMLS	0.54 (+0.01)	0.53 (+0.00)	0.53 (+0.00)	0.55 (+0.00)	0.52 (+0.01)
**ORDO-SNOMED CT**					
Precision BioPortal	0.30 (+0.01)	0.35 (+0.00)	0.36 (+0.01)	0.30 (+0.00)	0.34 (+0.01)
Precision UMLS	0.49 (+0.01)	0.5 (+0.00)	0.50 (+0.01)	0.50 (+0.01)	0.50 (+0.01)
F1-score BioPortal	0.44 (+0.00)	0.49 (+0.00)	0.50 (+0.00)	0.44 (+0.00)	0.47 (+0.00)
F1-score UMLS	0.57 (+0.01)	0.51 (+0.00)	0.52 (+0.00)	0.56 (+0.01)	0.52 (+0.00)
